# Supplementary figures and images for: Co-expression network analysis reveals the pivotal role of mitochondrial dysfunction and interferon signature in juvenile dermatomyositis
Source: PeerJ. 2020 Feb 18;8:e8611. doi: 10.7717/peerj.8611 (PMC7034382; doi:10.7717/peerj.8611)

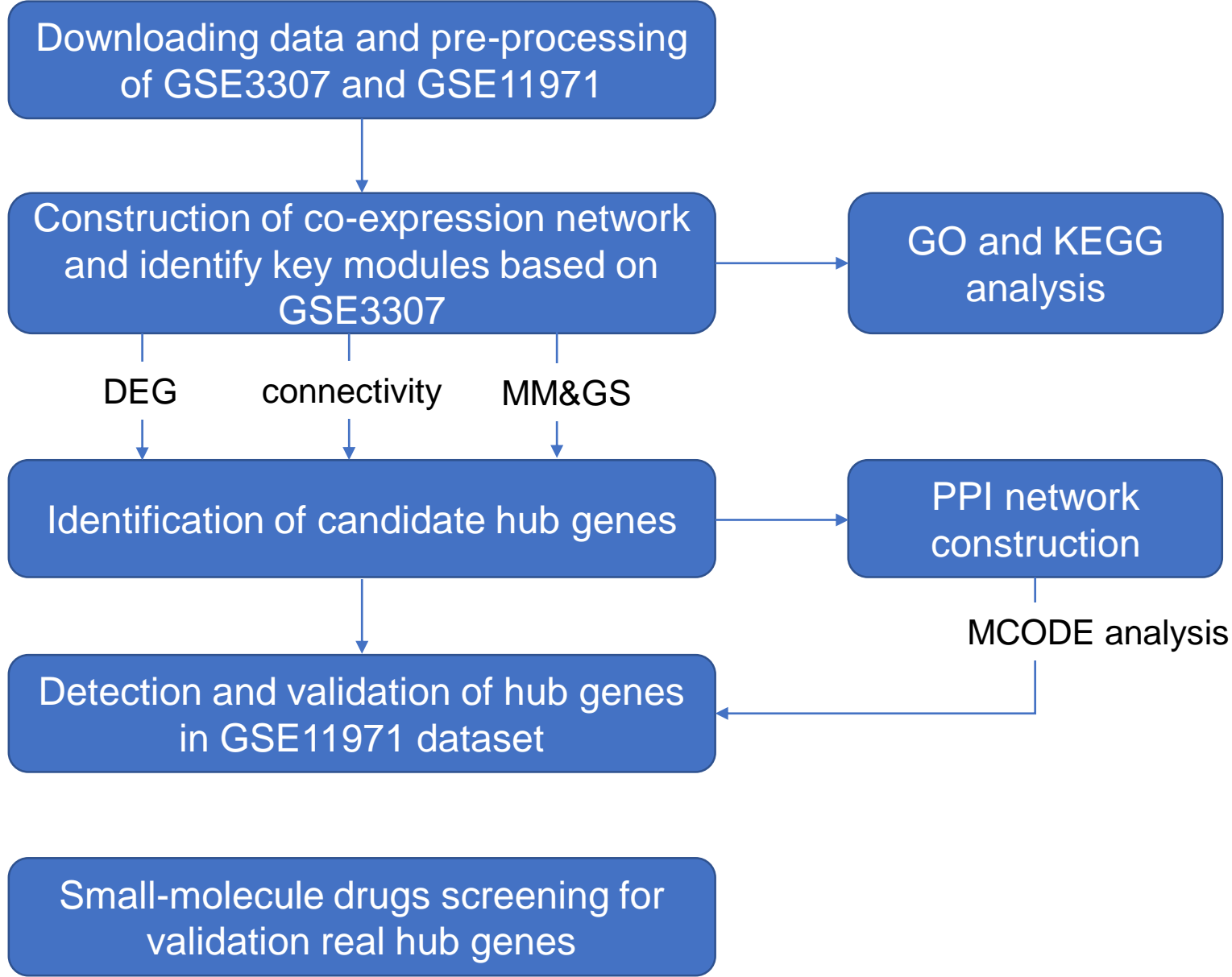

Supplement: Figure S1 [file peerj-08-8611-s004.pdf]

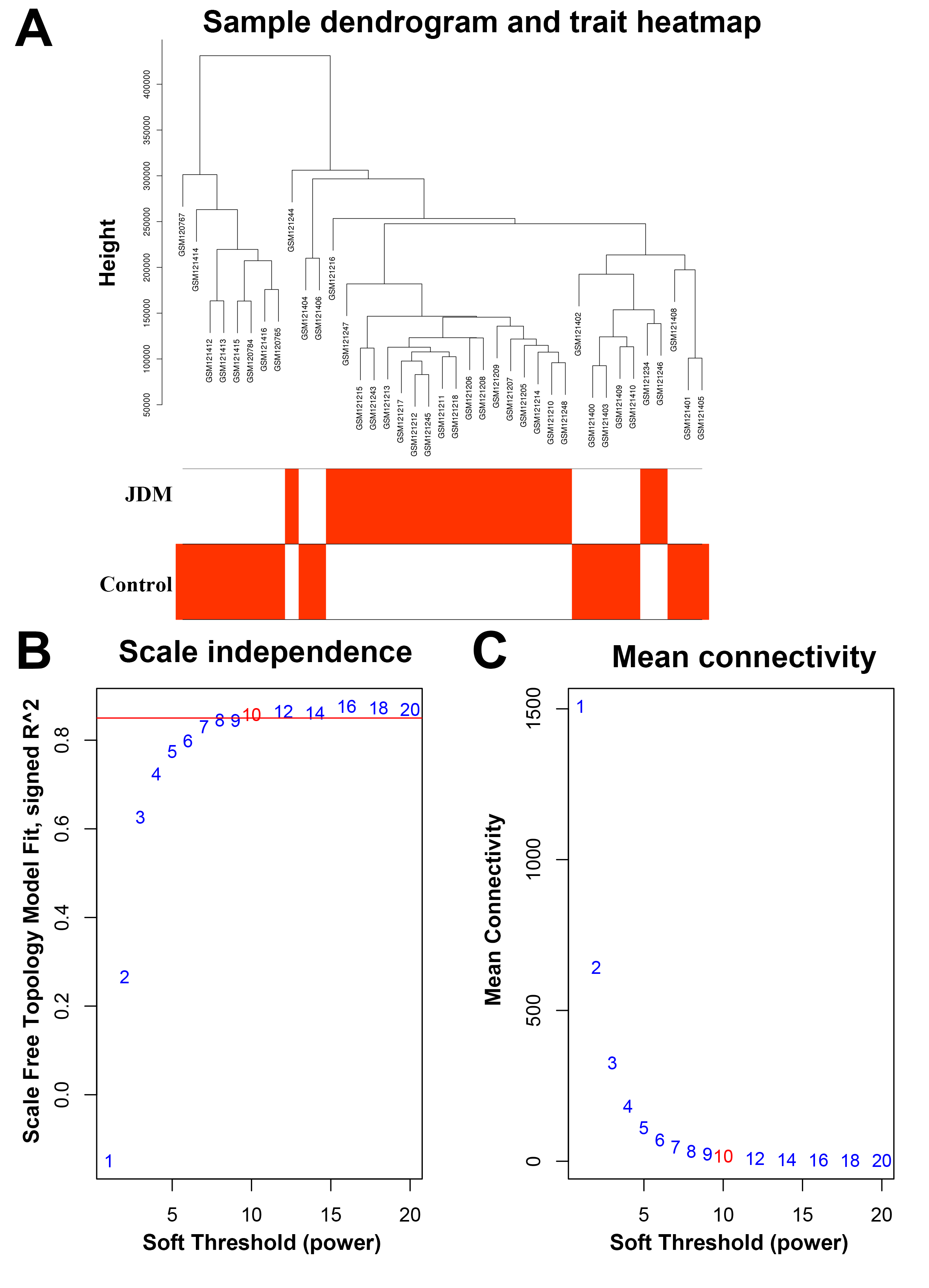

Supplement: Figure S2 — (A) Sample clustering was conducted to detect outliers. All samples are located in the clusters and pass the cutoff thresholds. (B) Analysis of the scale-free fit index for various soft-thresholding powers (β). (C) Analysis of the mean connectivity for various soft-thresholding powers. [file peerj-08-8611-s005.png]

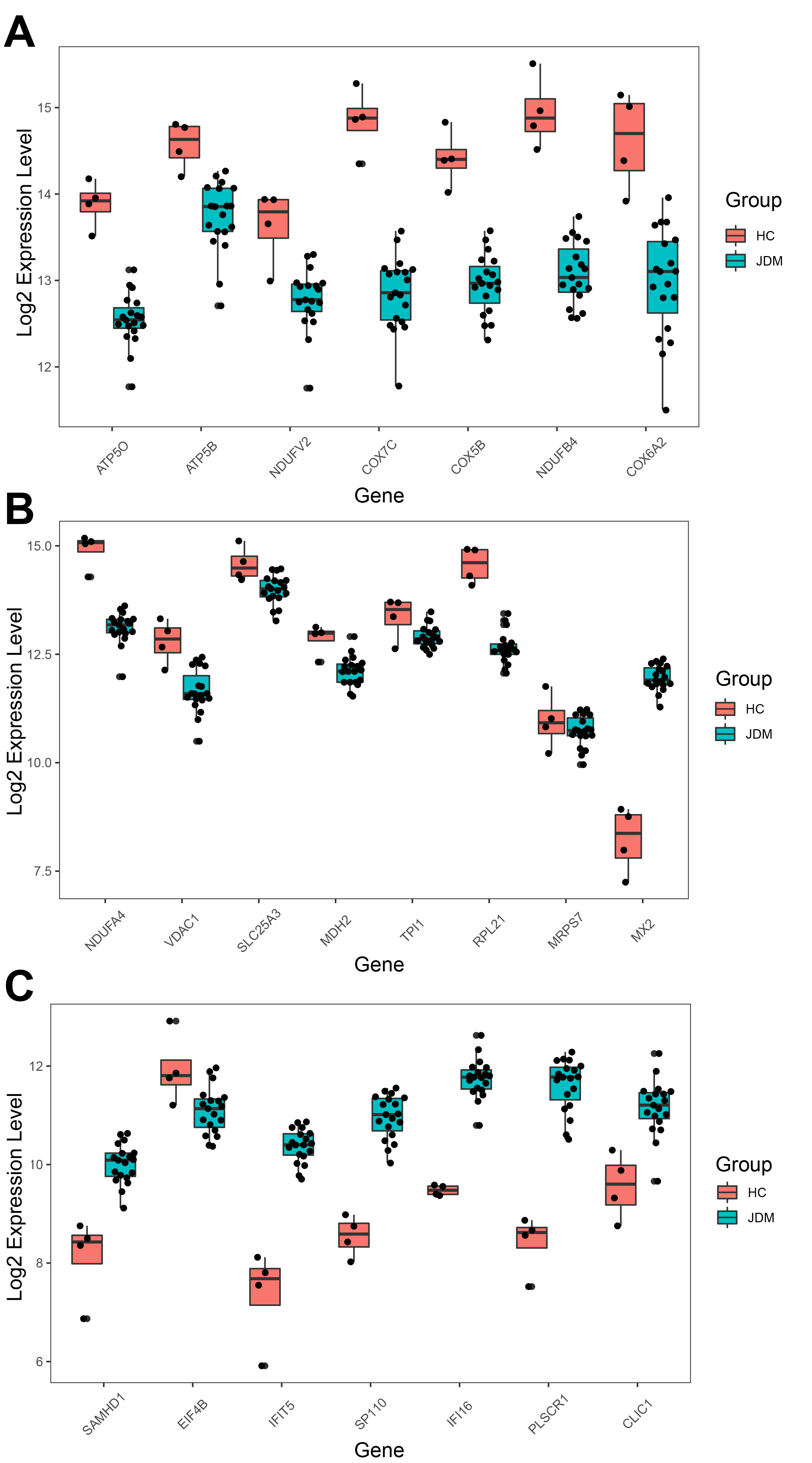

Supplement: Figure S3 — (A) ATP5O, ATP5B, NDUFV2, COX7C, COX5B, NDUFB4 and COX6A2; (B) NDUFA4, VDAC1, SLC25A3, MDH2, TPI1, RPL21, MRPS7 and MX2; (C) SAMHD1, EIF4B, IFIT5, SP110, IFI16, PLSCR1, and CLIC1. [file peerj-08-8611-s006.png]
